# From the Gender Gap to Neuroactive Steroids: Exploring Multiple Cases to Further Understand Neuropathic Pain

**DOI:** 10.3390/ijms24108577

**Published:** 2023-05-11

**Authors:** Sara Marinelli, Roberto Coccurello

**Affiliations:** 1 National Council of Research (CNR), Institute of Biochemistry and Cell Biology, 00015 Monterotondo, Italy; 2 Institute for Complex Systems (ISC), National Council of Research (CNR), 00185 Rome, Italy; 3 European Center for Brain Research/Santa Lucia Foundation IRCCS, 00143 Rome, Italy

Neuropathic pain (NeuP) is still an intractable form of highly debilitating chronic pain, resulting from a lesion or disease of the somatosensory nervous system [1]. It is well-accepted that the pharmacological management of NeuP is still an unsolved challenge, and that peripheral NeuP can only in part be alleviated via the administration of a variety of drugs and drug combinations including antidepressants that inhibit norepinephrine and serotonin reuptake (e.g., duloxetine), opioid-based analgesics (e.g., tramadol), gabapentinoid drugs such as ligands of the *α* _2_*δ*-1 and *α* _2_*δ*-2 auxiliary subunits of calcium channels (e.g., gabapentin and pregabalin) and local therapy with lidocaine or capsaicin patches and/or injections of botulinum toxin type A [2,3].

The scientific contributions enclosed in the Special Issue “The Multiple Mechanisms Underlying Neuropathic Pain” reflect the complexity of the current scenario by providing compelling examples of the different approaches to the investigation of mechanisms and the development of potential therapies for the control of NeuP. In this regard, emblematic are the two papers by Chang C. et al. and Basu P. and colleagues entitled “Cross-Talk of Toll-Like Receptor 5 and Mu-Opioid Receptor Attenuates Chronic Constriction Injury-Induced Mechanical Hyperalgesia through a Protein Kinase C Alpha-Dependent Signaling” [4] and “Effects of Curcumin and Its Different Formulations in Preclinical and Clinical Studies of Peripheral Neuropathic and Postoperative Pain: A Comprehensive Review” [5], respectively. The first paper is a scientific investigation that analyzes the molecular underpinnings of the cross-talk between member 5 of the toll-like receptors (TLR5) and the mu-opioid receptor (MOR) that was found to be implicated in the process of nerve decompression following chronic constriction injury (CCI) of the sciatic nerve. Indeed, nerve decompression increased TLR5 expression at the DRG neurons of lamina II and, at the same level, the parallel overexpression of MORs. TLRs are recognized as having a major role in innate immunity (e.g., PAMP recognition) and in inflammatory responses, and TLR-mediated signaling has previously been demonstrated in pain mechanisms, especially for TLR2, TLR3, and TLR4 [6,7]. Recently, emerging attention has also been focused on TLR3, TLR5, TLR7 and TLR9 members [8,9], and the work by Chang C. et al. can be considered useful for gaining further insight into TLR5-mediated signaling, nociception regulation, and the modulation of sensory neuropathy. The authors provide additional evidence of an underlying mechanism whereby the activation of the protein kinase C isoenzyme α (PKCα) mediates the functional interaction between MOR and TLR5, which provides an indication for the future development of analgesic drugs. However, the development of therapeutics against NeP has also been involved in the robust trajectory of research focused on natural products and their capacity to display a bioactive role and exert an analgesic action. Indeed, conversely, the contribution of Basu P. and colleagues consists of a review paper aiming at describing the “state of the art” of polyphenolic compounds, and particularly of Curcumin use, as well as Curcumin-derived formulations, to clinically manage or mitigate NeP. An analysis of the literature is accomplished by considering the potential efficacy of Curcumin treatment in a variety of different neuropathies such as alcoholic neuropathy, chemotherapy-induced peripheral neuropathy, diabetic painful neuropathy and peripheral NeP induced by peripheral nerve injury. Of special interest is the summary concerning the effects achieved via curcumin and curcuminoid administration in clinical settings, showing an interesting degree of reported efficacy in different conditions such as diabetic neuropathy, peripheral neuropathy and post-operative pain. The formulations used in these clinical reports were, however, quite dissimilar to each other, ranging from nano-curcumin formulations and lecithinized curcumin, to a combination of curcumin and amoxicillin. In general, these reports suggest that the use of highly concentrated and bioavailable Curcumin formulae can lead to a reduction in dosage of common non-steroidal anti-inflammatory drugs such as dexibuprofen. Moreover, some electrophysiological and molecular changes induced via Curcumin administration are schematized, thus providing the opportunity for a further, future exploration of the potential overlap between mechanisms involved in the antinociceptive and anti-allodynic effects produced by novel drugs (e.g., those acting on TLR-signaling pathways) and the mechanisms implicated in the biological activity of some flavonoids and polyphenols, including Curcumin and curcuminoids (i.e., turmeric derivatives).

This first volume of the Special Issue “The Multiple Mechanisms Underlying Neuropathic Pain” also contains also two additional reviews; the first concerns the role of the insular cortex (ICx) in NeP [10] while, to the second concerns the role of neuroactive steroids in peripheral neuropathy as a factor underlying the sexual dimorphism of pain sensitivity [11]. Herein, we present two different approaches with which to investigate the elusive problem of NeP that for the study of the role of ICx consider the alteration in neural plasticity as a potential underlying mechanism whereby the initial stage of acute pain is converted to pharmacologically intractable chronic pain. The contribution of ICx to pain processing is likely due partially to the fact that pain is a very subjective experience in which there is an important affective-motivational component, and that ICx is highly interconnected with the limbic system. Thus, the study examines several aspects in which ICx and pain processing are associated, starting with the morphological and functional changes that can be detected during the progression of neuropathy. Since the activation of ICx is involved in the generation of pain and correlates with the intensity of noxious stimuli [12,13], there is a rationale for hypothesizing that different classes of analgesic drugs may have a key anatomic site in the ICx by which their mechanisms of action and/or efficacy can be explained. Indeed, in their paper, Wang and al. [10] addressed these mechanisms by illustrating the distribution of opioid, endocannabinoid, dopaminergic, glutamatergic and oxytocin receptors within the ICx, also reporting a plethora of evidence of their role in ICx-dependent pain processing. However, the ICx can also be considered a non-pharmacological site for therapeutic intervention [14] as well as for the investigation of NeP comorbidities such as depression [15]. Quite interestingly, sex differences and different patterns of cortical plasticity (e.g., in the anterior cingulate cortex) have been described in male and female individuals [16,17]; thus, they may influence pain perception and emotional responses to noxious stimuli. The review study by Falvo and colleagues [11] deals with damage to the peripheral nervous system (PNS) and the ability of steroids synthetized by both the central nervous system and steroidogenesis in the PNS to regulate neural transmission and myelin function. Since levels of neuroactive steroids such as pregnenolone, dihydroprogesterone and tetrahydroprogesterone in brain areas (e.g., the cortex, hippocampus and cerebellum) and the peripheral nerves are also different among the two sexes, their gender-dependent secretion directly affects pain processing and pain perception/sensitivity in male and female subjects. Hence, novel analgesic therapies should take into account the fluctuations in sex steroids as these deserve dedicated attention toward the sex-dependent differences in the susceptibility to chronic pain and the capacity for recovery. Indeed, the last paper included in this Special Issue, “The Multiple Mechanisms Underlying Neuropathic Pain”, is a research article by Vacca et al. [18] that investigates gender differences in the responses to peripheral nerve lesion, contributing to the body of evidence on the different immune and neuroimmune profiles underlying chronic NeP in male and female mice. Some immune cell populations were found to be differently expressed, such as, for instance, the higher T cell infiltration in injured nerves from female animals. Interestingly, leptin levels were upregulated in sciatic nerves from female mice, thus partially accounting not only for the greater pain sensitivity and development of chronic NeP (e.g., inflammation and a higher proliferation of spinal microglia), but also indicating a mechanism whereby alterations of adipocytokines secondary to the dysfunction of adipose tissue may interact with changes in neuroendocrine function and, in turn, steroid hormones modulate leptin secretion and pain susceptibility in a sex-dependent manner.
